# Tau seeding without tauopathy

**DOI:** 10.1016/j.jbc.2023.105545

**Published:** 2023-12-09

**Authors:** Michael S. LaCroix, Efrosini Artikis, Brian D. Hitt, Joshua D. Beaver, Sandi-Jo Estill-Terpack, Kelly Gleason, Carol A. Tamminga, Bret M. Evers, Charles L. White, Byron Caughey, Marc I. Diamond

**Affiliations:** 1 Center for Alzheimer’s and Neurodegenerative Diseases, Peter O’Donnell Jr. Brain Institute, University of Texas Southwestern Medical Center, Dallas, Texas, USA; 2 NIH/NIAID, Rocky Mountain Laboratories, Hamilton, Montana, USA; 3 Department of Neurology, UT Southwestern Medical Center, Dallas, Texas, USA; 4 Department of Psychiatry, UT Southwestern Medical Center, Dallas, Texas, USA; 5 Department of Pathology, UT Southwestern Medical Center, Dallas, Texas, USA

**Keywords:** Alzheimer’s disease, tauopathy, FRET biosensor, prion, tau seeding activity, healthy brain

## Abstract

Neurodegenerative tauopathies such as Alzheimer’s disease (AD) are caused by brain accumulation of tau assemblies. Evidence suggests tau functions as a prion, and cells and animals can efficiently propagate unique, transmissible tau pathologies. This suggests a dedicated cellular replication machinery, potentially reflecting a normal physiologic function for tau seeds. Consequently, we hypothesized that healthy control brains would contain seeding activity. We have recently developed a novel monoclonal antibody (MD3.1) specific for tau seeds. We used this antibody to immunopurify tau from the parietal and cerebellar cortices of 19 healthy subjects without any neuropathology, ranging 19 to 65 years. We detected seeding in lysates from the parietal cortex, but not in the cerebellum. We also detected no seeding in brain homogenates from wildtype or human tau knockin mice, suggesting that cellular/genetic context dictates development of seed-competent tau. Seeding did not correlate with subject age or brain tau levels. We confirmed our essential findings using an orthogonal assay, real-time quaking-induced conversion, which amplifies tau seeds *in vitro*. Dot blot analyses revealed no AT8 immunoreactivity above background levels in parietal and cerebellar extracts and ∼1/100 of that present in AD. Based on binding to a panel of antibodies, the conformational characteristics of control seeds differed from AD, suggesting a unique underlying assembly, or structural ensemble. Tau’s ability to adopt self-replicating conformations under nonpathogenic conditions may reflect a normal function that goes awry in disease states.

Accumulation of intracellular assemblies of the microtubule-associated protein tau (MAPT) underlies myriad neurodegenerative diseases termed tauopathies (1). Alzheimer’s disease (AD), the most common tauopathy, is estimated to afflict ∼50 million people worldwide, and ∼150 million by 2050 (https://www.alzint.org/resource/numbers-of-people-with-dementia-worldwide/). Most tauopathies are sporadic, while some are caused by dominantly inherited mutations in the MAPT gene (1). The origin of sporadic tauopathies has remained elusive, but growing experimental evidence suggests that tau functions as a prion in pathological states. Initial work from our group and others has suggested that tau stably propagates unique fibrillar structures *in vitro* (2). In living systems, exogenous tau assemblies are spontaneously taken up by cultured cells and serve as templates for intracellular aggregation (3), and, similarly, tau inoculated into transgenic mouse brain induces intracellular pathology (4). Diverse neuropathologies also form in a tauopathy mouse model upon brain inoculation with homegenates prepared from human tauopathies (5). We have further observed that tau forms a variety of unique structures, or strains, that propagate indefinitely and transmit readily among cells, and, after inoculation into a mouse model, produce unique, transmissible patterns of neuropathology (6). We also observed unique strain composition patterns in five different tauopathies, including variation within specific neuropathological diagnoses (6). In a large survey of 18 strains propagated in cells, we determined that each gave rise to a unique pattern of neuropathology following inoculation into a mouse model (7). This work has been confirmed and extended by cryo-electron microscopy (cryo-EM) of tau fibril structures extracted from different tauopathies, which has revealed unique core structural features that correlate with different neuropathological diagnoses (8, 9, 10, 11). Recent work reports fibril diversity within a single neuropathological diagnosis in multiple tauopathies (12), consistent with our prior isolation of distinct strains from individual brains (6). Taken together, considerable evidence supports the idea that pathological assemblies of distinct structure drive the development of unique tauopathies.

The faithful maintenance of tau strains in cells, mice, and humans suggests the existence of an intrinsic replication machinery that participates in the amplification of unique structures. Meanwhile, the normal function of tau is not entirely clear. Tau binds microtubule filaments (13, 14) through interactions across the interface of tubulin heterodimers (15, 16) and has been proposed to stabilize microtubules *in vivo* (17, 18). Yet tau knockout mice are viable (19), suggesting microtubule stabilization is not an essential function, or that it is replicated by other proteins.

An enormous literature suggests that many proteins form self-amplifying assemblies to regulate biological processes. The first examples were described in yeast (20), and similar protein activities in mammals are now established. For example, the prion-like polymerization of MAVS and ASC proteins transduces signaling in innate immunity and inflammation based on self-propagating assemblies (21, 22). In mice, the cytoplasmic polyadenylation element-binding protein changes from a soluble to an aggregated state that promotes translation of sequestered synaptic mRNAs, maintaining long-term potentiation (23, 24, 25, 26, 27). Last, in hippocampi of mice exposed to cellular stress, TIA-1, which facilitates the assembly of stress granules, forms heritable aggregates through its amyloidogenic C-terminal prion-like domain (26). Given data that tau functions as a prion in experimental systems, we hypothesized a physiological role for tau conformers that serve as templates for their own replication, *i.e.*, “seeds.” According to this model, tau aggregation in disease represents a normally occurring process that might escape physiologic regulation, not a *de novo* and purely pathological function. This model predicts the existence of tau seeds in healthy individuals, but until now they have eluded detection except as weak and poorly understood signals in tau-RT-QuIC *in vitro* seed amplification assays (35, 36). In this study we use immunoprecipitation (IP) of brain lysate with a novel anti-tau antibody, a highly sensitive cell-based biosensor assay (28), an RT-QuIC assay, and immunoassays to address this question.

## Results

### Tau seeding in parietal cortex

In preliminary work we screened available antibodies for those that would most efficiently precipitate seeding activity from control brain and identified MD3.1, an antibody raised against a peptide of R1/R3 of the tau repeat domain (aa263–311 Δ275-305) with a “trans” proline residue consisting of N-Boc-trans-4-fluoro-L-proline substituted at P270. MD3.1 binds tau seeds with high efficiency (29). We then used MD3.1 to test brain lysates from 19 control subjects of diverse ages (19–65 years) (Table 1).

**Table 1:** Demographic Data from the cases studied

Case	Sex	Age (years)	Race	Diagnoses	PMI (hrs)	Manner and cause of death
A	M	20	C	Control	21.2	Accident—chest compression from blunt force injuries
B	M	47	C	Control	24	Natural—cardiovascular disease and diabetes
C	M	34	C	Control	23.4	Natural—cardiovascular disease and diabetes
D	M	52	C	Control	24	Accident—electrocution
E	F	45	C	Control	22.5	Natural—cardiovascular disease
F	M	29	C	Control	27.4	Natural—polycystic kidney disease
G	F	50	C	Control	23.3	Natural—adrenocortical deficiency
H	F	62	C	Control	19.4	Natural—chronic alcoholism
I	M	48	C	Control	14.7	Natural—mitral valve regurgitation
J	M	54	C	Control	19.3	Natural—cardiovascular disease
K	M	19	C	Control	20	Homicide—gunshot wound
L	M	36	C	Control	23	Undetermined
M	M	34	C	Control	24	Accident—drowning
N	F	49	PI	Control	15.3	Natural—cardiovascular disease
O	F	65	C	Control	11	Natural—cardiovascular disease
P	M	65	C	Control	14	Natural—respiratory arrest
Q	M	63	C	Control	14	Natural—myocardial infarction
R	F	55	C	Control	25	Natural—intracerebral hemorrhage
S	M	32	C	Control	21.5	Natural—pulmonary embolism

The 19 tauopathy-negative control cases studied span 6 decades of life ranging in age from 19 to 65 years. C, Caucasian; F, female; M, male; PI, Pacific Islander; PMI, postmortem interval.

**Figure 1:**
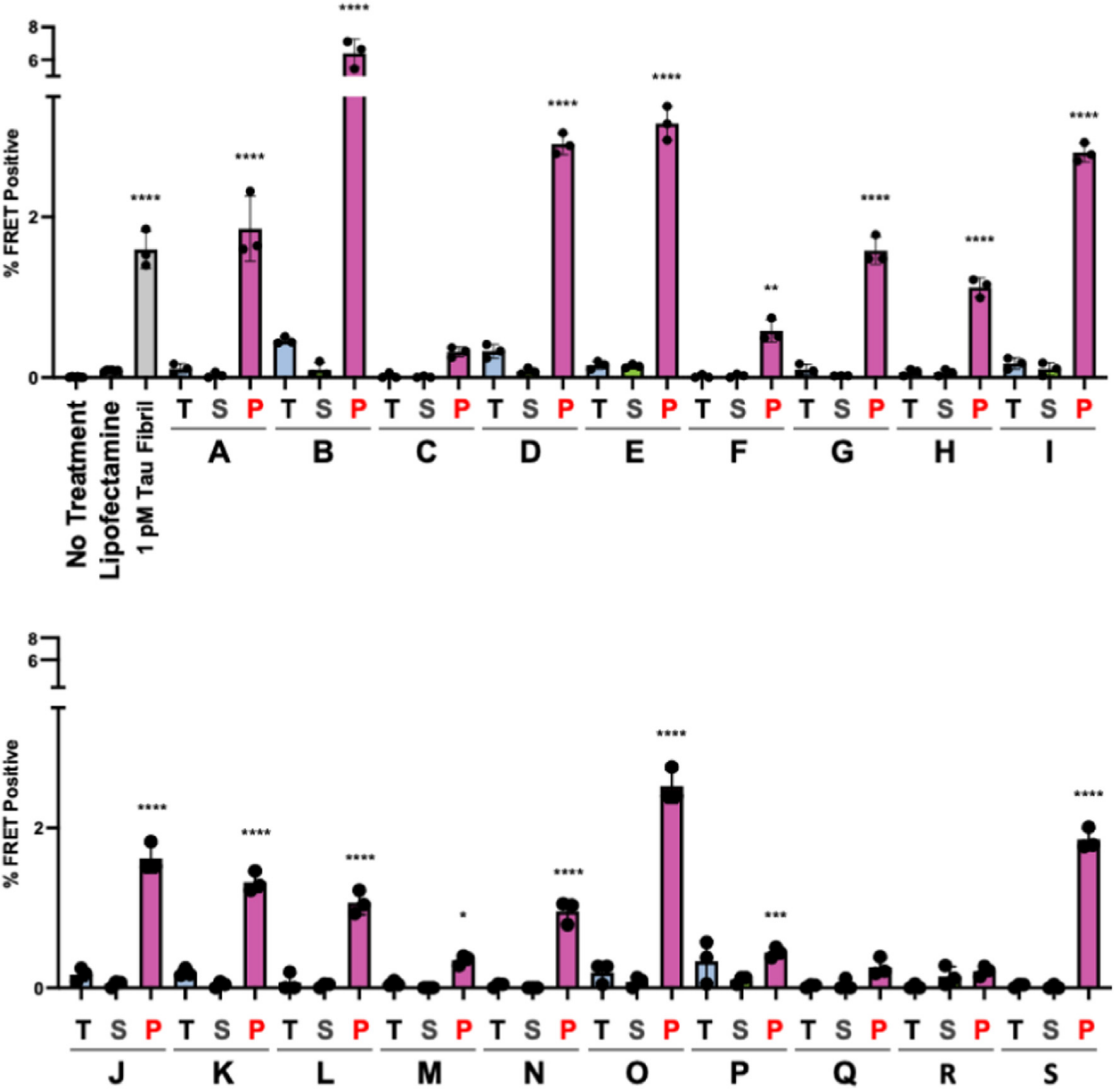
**Tau seeding is present in the parietal lobe of 19 control subjects.** Parietal cortex fresh-frozen samples from 19 individuals (*A*–*S*) without any known neurological diagnoses were used to create total (T) clarified lysate (10% [wt/vol]) followed by immunoprecipitation with the MD3.1 antibody to generate a tau-depleted supernatant (S) and tau-enriched pellet (P). Tau seeding was reliably detected in 16 of 19 cortical immunoprecipitation pellets. Columns represent the mean fluorescence resonance energy transfer (FRET) positivity from three technical replicates (*dots*). Statistical significance was determined by performing one-way ANOVA followed by Dunnett’s multiple comparisons testing of all samples compared against Lipofectamine-treated negative controls, ∗*p* < 0.05, ∗∗*p* < 0.01, ∗∗∗*p* < 0.001, ∗∗∗∗*p* < 0.0001. Error bars = SD.

We prepared soluble protein lysates from fresh-frozen tissue of the cortex of the parietal lobe and immunoprecipitated the total soluble protein lysate using MD3.1 antibody. We then used Lipofectamine 2000 to deliver total protein lysate (T), tau-depleted IP supernatants (S), and tau-enriched IP pellets (P) into v2H tau biosensors (28). Nontreated and Lipofectamine-treated cells were used as negative controls; recombinant heparin-derived 2N4R tau fibrils (1 pM monomer equivalent) were used as a positive control. All tau-enriched IP pellets exhibited seeding activity levels beyond that of Lipofectamine-treated controls, with 16/19 tau-enriched IP pellets reaching statistical significance (*p* < 0.05) compared with Lipofectamine-treated controls when tested by ANOVA (Fig. 1). MD3.1 did not induce a transition from inert to seed-competent tau when tested with recombinant tau monomer (Fig. S1). We concluded that human cortical tissue contains tau seeding activity, in the absence of known tauopathy.

### Tau seeding is absent in the cerebellum

**Figure 2:**
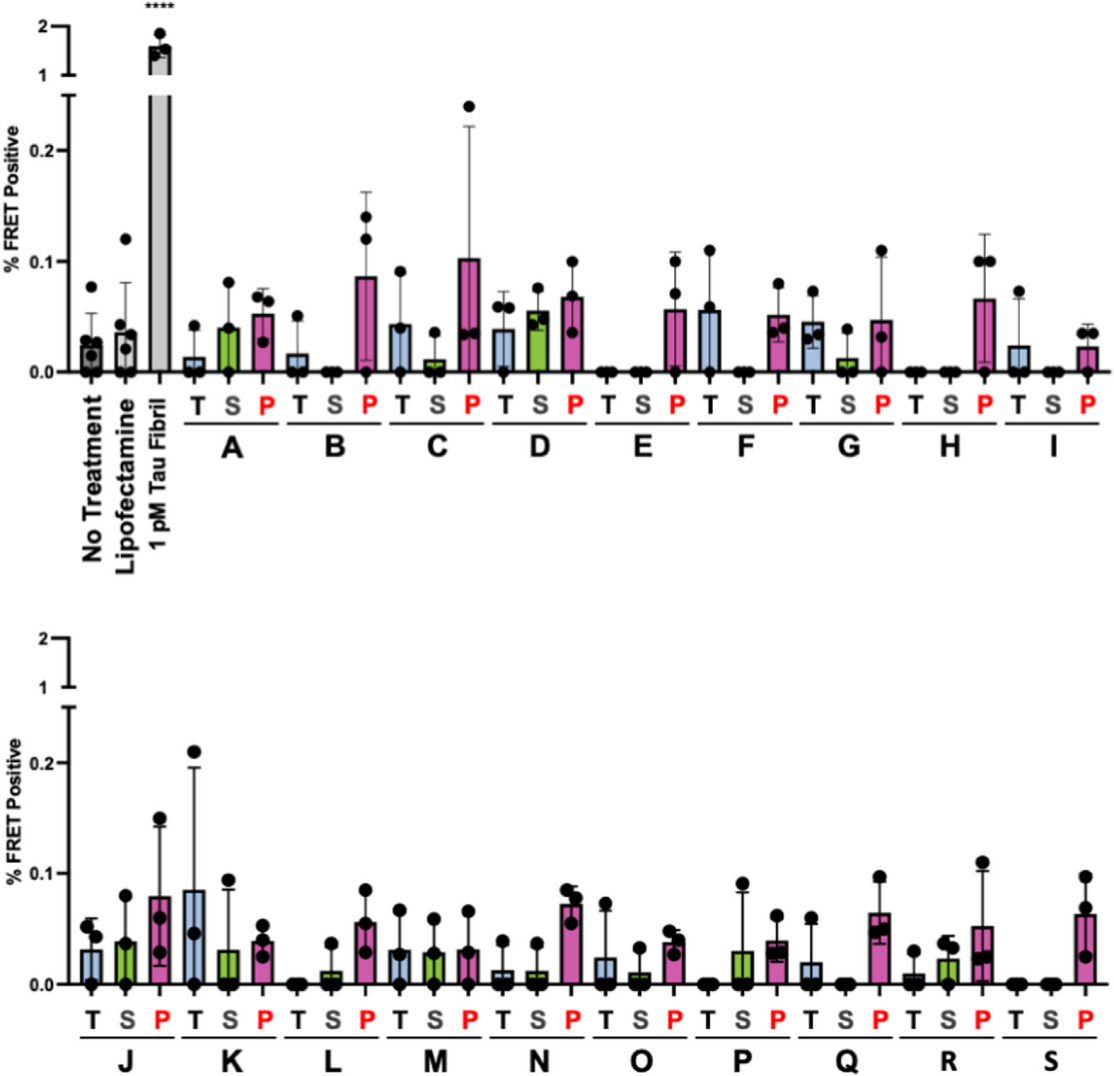
**No reliable tau seeding activity is detected in the cerebellum of 19 control subjects.** Fresh-frozen cerebellum samples from 19 subjects without any known neurological diagnoses (referenced *A*–*S*) were used to create total (T) clarified lysate (10% [wt/vol]) followed by immunoprecipitation with the MD3.1 antibody to generate a tau-depleted supernatant (S) and tau-enriched pellet (P). No reliable tau seeding was detected across all cerebellum samples. Columns indicate the mean fluorescence resonance energy transfer (FRET) positivity from three technical replicates (*dots*). Statistical significance was determined by performing one-way ANOVA followed by Dunnett’s multiple comparisons testing of all samples compared against Lipofectamine-treated negative controls, ∗∗∗∗*p* < 0.0001. Error bars = SD. Note alteration of seeding scale to encompass the positive control.

A past study found that tau seeding is largely absent from the cerebellum except at late Braak stages (30). Consequently, we tested for seeding activity in the cerebellum samples. We prepared total soluble protein lysates and performed IP with the MD3.1 antibody. We did not detect significant seeding in any sample, although we could not exclude the presence of very low levels of seeds enriched by IP (Fig. 2).

### Tau seeding is absent in wildtype and hTau knockin mice

**Figure 3:**
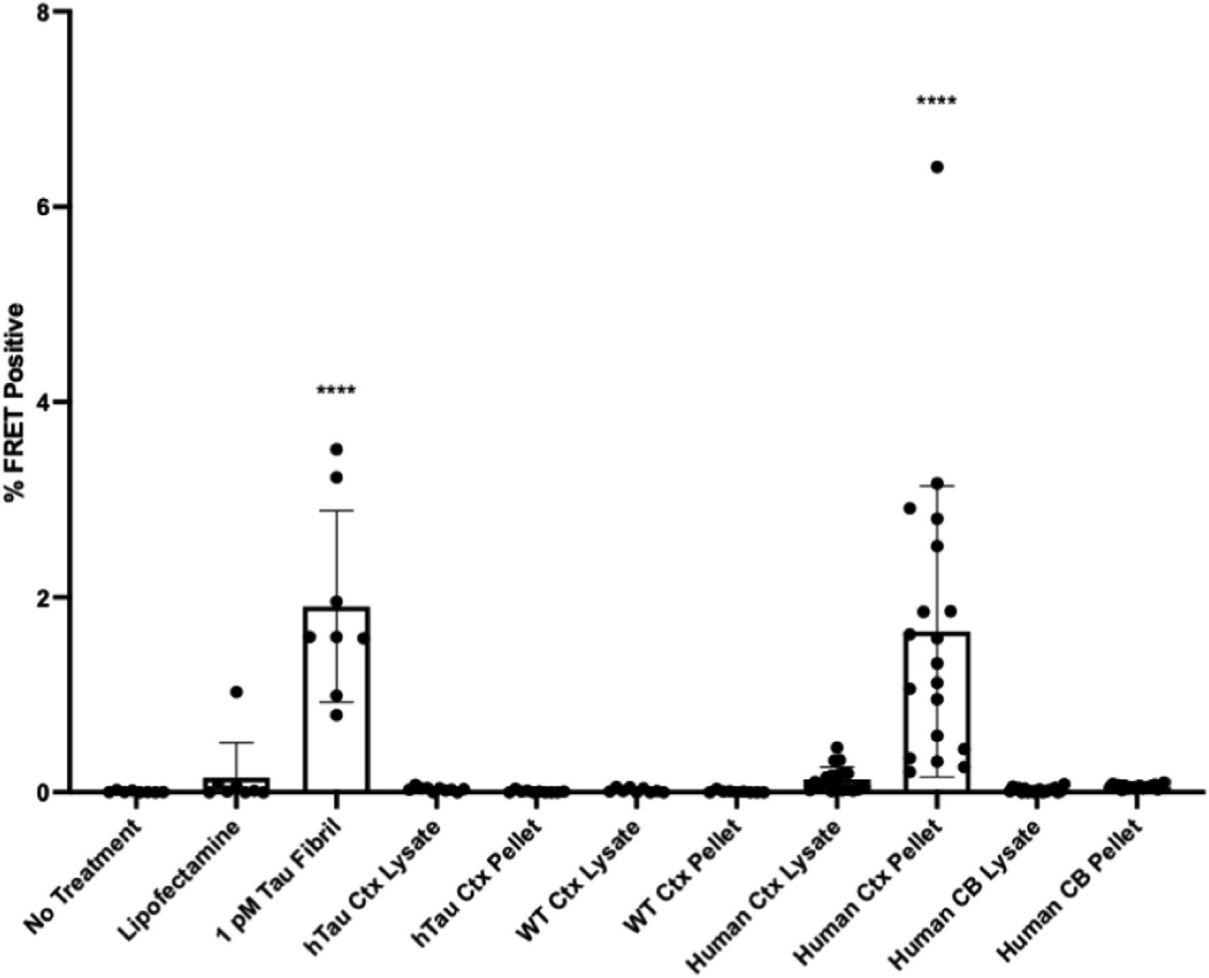
**Human and mouse tau expressed in mouse cortex does not form detectable seeds.** Of the groups tested, only human tau enriched *via* immunoprecipitation from human cortex, and not human cerebellum, formed seeds that were detectable at a statistically significant level. Tau immunoprecipitated from the cortex of mice expressing human tau (hTau, n = 10, F = 5, M = 5) and wildtype mouse tau (WT, n = 9, M = 4, F = 5) did not show significant seeding activity. Statistical significance was determined by performing one-way ANOVA followed by Dunnett’s multiple comparisons testing of all samples compared against Lipofectamine-treated negative controls, ∗∗∗∗*p* < 0.0001. Error bars = SD. CB, cerebellum; Ctx, cortex; FRET, fluorescence resonance energy transfer.

The low or absent tau seeding in the cerebellum suggested that its development might be cell type dependent. Human tau expressed in a knockin mouse provided the perfect opportunity to extend this inquiry. These mice (a gift from Pfizer) express all six isoforms of human tau under the mouse promoter. We first confirmed that these animals express human tau by using Western blot with HJ8.5, a monoclonal antibody specific for human tau (31). We prepared total protein lysates from the brains of adult human tau knockin mice (hTau), BL6/C3H wildtype (WT), and tau knockout mice. We detected tau in knockin mouse brain with HJ8.5, which did not detect mouse tau in WT mice (Fig. S2*A*). By contrast, a polyclonal anti-tau antibody (A0024, DAKO) that detects mouse and human tau revealed tau protein in hTau and WT mice (Fig. S2*B*). We used IP with MD3.1 to test for seeding in the mouse brains, with human samples as a positive control. We detected no significant seeding activity in any mouse-derived samples, including the tau-enriched IP pellet (Fig. 3). We concluded that tau expression in a mouse cortical neuron was not sufficient to induce a seed-competent form and other factors must be required.

### RT-QuIC detects tau seeds *in vitro*

Tau biosensor cells have documented specificity for tau seeds (32). Nonetheless, to confirm our findings, we also analyzed samples with an orthogonal, cell-free system. Real-time quaking-induced conversion (RT-QuIC) is a well-established assay that has been used to detect pathological seeds derived from the prion protein, α-synuclein, and tau (33). RT-QuIC exposes test samples to high concentrations of tau monomer, and through repeated cycles of shaking and fibril growth *in vitro*, the time to form detectable fibrils is determined based on thioflavin fluorescence. This method is highly sensitive and specific (34, 35, 36, 37) and reports the lower limit of detection and seed titer through serial dilutions (38). Our recent studies had detected tau seeding in the brains of tauopathy cases and much lower levels in many control samples without known tauopathy that were of uncertain significance at the time (33, 35, 36).

**Figure 4:**
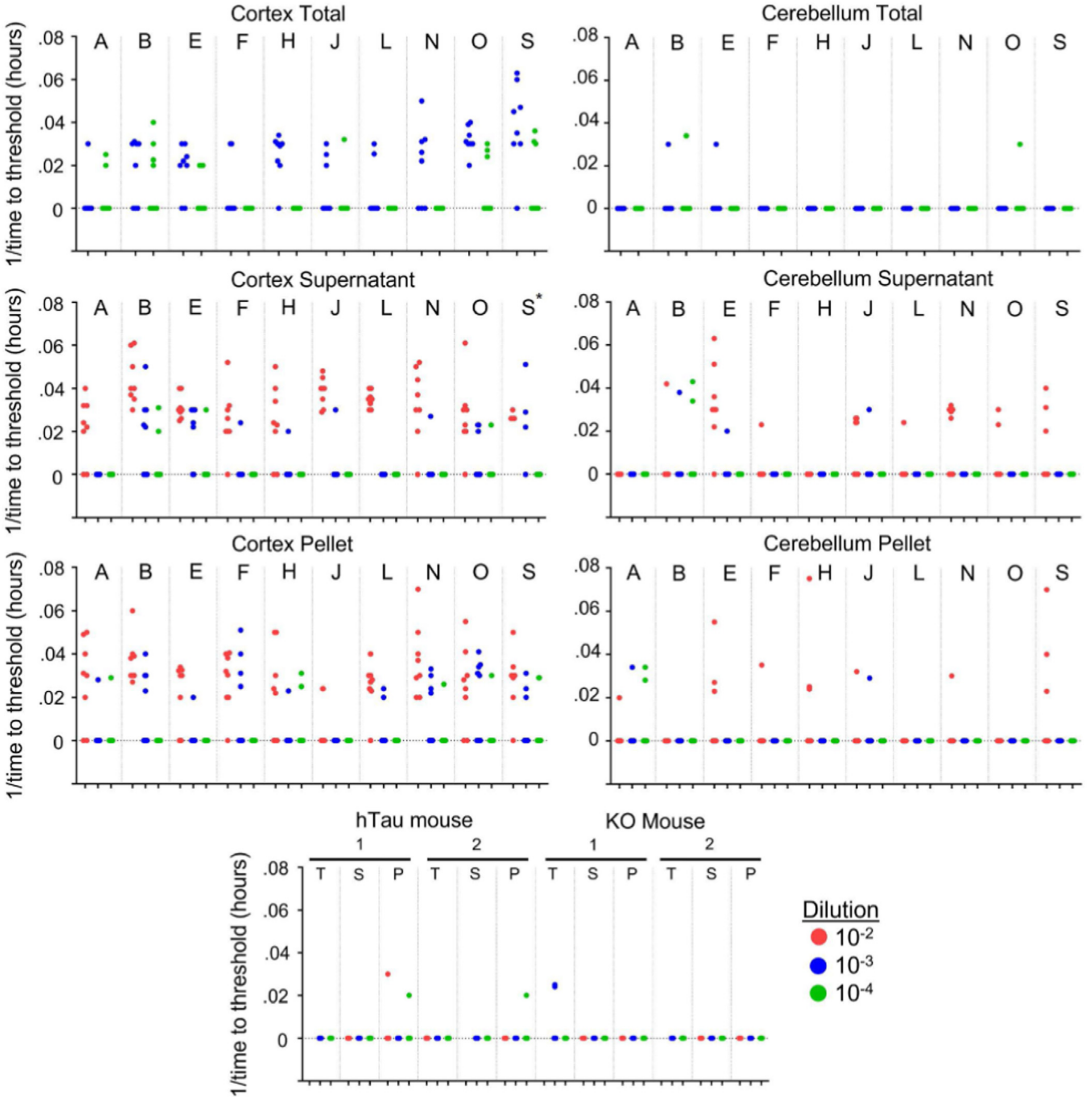
**RT-QuIC detects tau seeds in control brain.** Total homogenates (Total), supernatants (Supernatant), and immunoprecipitated pellets (Pellet) were diluted in KO/N2/Hepes buffer as indicated: 10-2, 10-3, 10-4 and used to seed the K12 RT-QuIC assay. The reciprocal of time to threshold thioflavin T fluorescence is plotted for eight replicate reactions except for sample marked S∗, which had only four replicates. Reactions for which 1/time to threshold values are marked as zero refer to those that failed to reach the threshold within 48 h. Nonzero data points are distinguishable as separate data points, whereas remaining replicates (out of a total of 8) are overlapped on the baseline. RT-QuIC, real-time quaking-induced conversion.

We tested the top 10 seeding samples identified with the biosensor assay, plus negative controls. We observed relatively weak seeding that was nonetheless positive for the sample overall by our usual criterion of having ≥50% of replicate reactions scoring above a fluorescence threshold (see Experimental procedures) in 6/10 total cortical homogenates, 9/10 supernatants, and 9/10 pellets. Using the same criteria, we observed much less seeding in cerebellum samples, with 0/10, 2/10, and 0/10 of the total, supernatant, and pellet samples, respectively, scoring positive, and always at the highest concentration tested (Fig. 4). Additionally, we detected only rare positive reactions seeded with hTau knockin or tau KO brain lysates (Fig. 4), which, in the latter case, must be due spontaneous nucleation events or inadvertent cross-contamination. The mean maximal thioflavin fluorescence for all samples, as expected, was lower than for sporadic AD specimens but was well above background (Fig. S3). Taken together, while the results were not identical to the biosensor system, they confirmed the essential findings of the presence of tau seeds in human control brains and absence in murine samples. The results effectively excluded the possibility that seed detection in the absence of tauopathy was due to “background” seeding within the biosensor cells.

### Control brains stain negative for AT8

**Figure 5:**
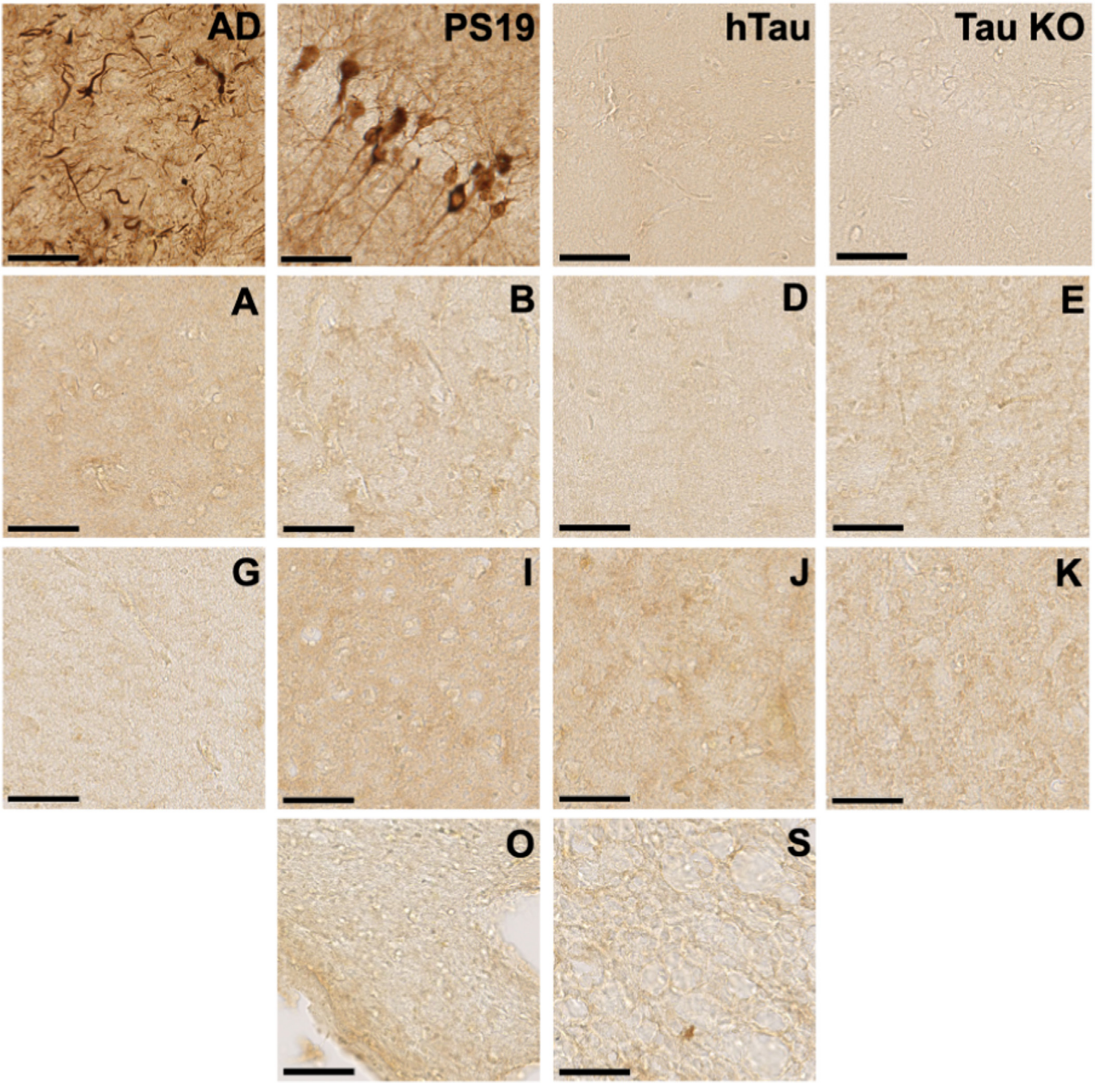
**Comparative AT8 staining of brain tissues.** Fresh-frozen brain samples were prepared from an Alzheimer’s disease (AD) control, an aged PS19 tauopathy mouse, and 10 control brain samples that had the highest levels of tau seeding and were imaged at 40× magnification (*A*, *B*, *D*, *E*, *G*, *I*–*K*, *O*, *S*). We easily detected tau pathology in AD and PS19 brain. We detected no AT8 signal in any control brain. The scale bar represents 50 μm.

To confirm that brains used for our studies did not harbor hidden tauopathy, we evaluated 10 brains with the highest seeding levels *via* immunohistochemistry with AT8. This mouse monoclonal antibody recognizes p-Ser202 and p-Thr205 and is an accepted standard for diagnosis of tauopathy (39). We selected brain regions as close as possible to those used for homogenates, typically within 1-2 cm. We observed no AT8 staining in any of the control brain samples, and, by contrast, we easily detected signal in AD and tauopathy mouse brain (Fig. 5).

**Figure 6:**
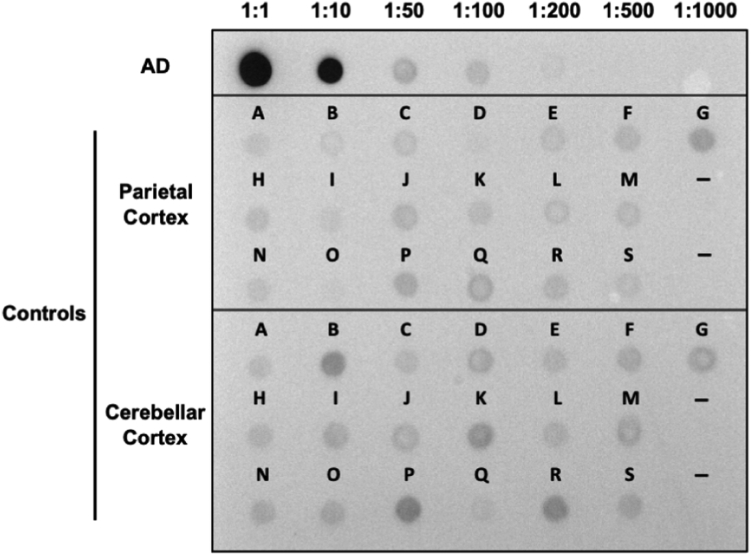
**Control brains contain markedly less AT8 positivity relative to Alzheimer’s disease (AD).** Two micrograms of soluble protein lysate from AD was used for 1:1, followed by serial dilutions. Two micrograms of total soluble protein was used for control samples, loaded in *left to right* order starting with sample A. Dashes represent no loading.

Because it was impossible to carry out histopathology on the precise brain regions studied by the seeding assay, we additionally analyzed homogenates for phospho-tau using AT8 to try to identify hidden tauopathy. We used a dot blot analysis to be sure that any larger assemblies would be fully detected, spotting 2 μg of brain lysate on nitrocellulose membrane for detection with AT8. We used an AD brain lysate in serial dilutions for reference (Fig. 6). AD lysate exhibited seeding activity approximately 10 to 100× more than was observed in all parietal and cerebellar samples from non-tauopathy brains. We found no difference in AT8 signal between parietal samples, which contained seeding activity, and cerebellar samples, which did not. Taken together, we concluded, based on biochemistry and immunohistochemistry that there was no evidence of increased AT8-positive tau within the brain samples we analyzed.

### Seeding is independent of total tau levels and subject age

**Figure 7:**
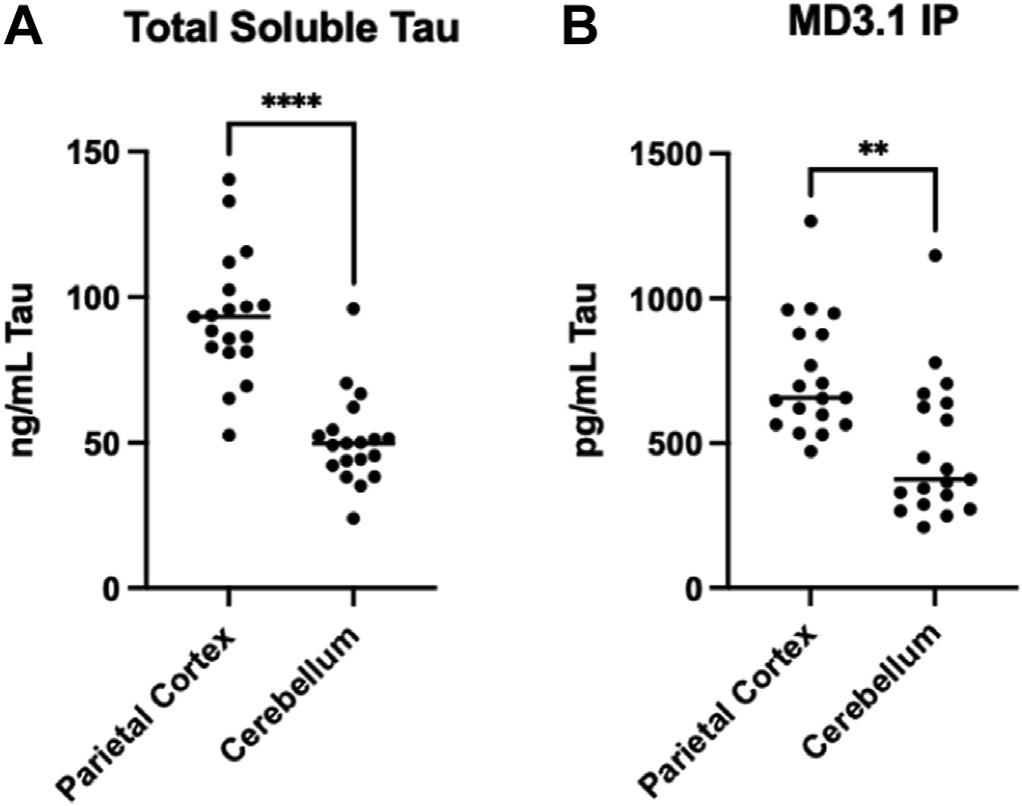
**ELISA quantification of tau in brain samples.***A*, quantification of soluble tau in total clarified lysates from the parietal cortex and cerebellum. *B*, quantification of tau in the pellet following immunoprecipitation using the MD3.1 antibody. Statistical significance was determined by performing Student’s *t* test, ∗∗*p* < 0.01, ∗∗∗∗*p* < 0.0001.

To test the correlation of tau levels and seeding activity, we used a tau ELISA developed by the Davies laboratory (40). Cerebellar protein lysates contained roughly half the tau of cortical lysates, although the ranges overlapped (Fig. 7*A*). Tau concentration in the cerebellar tau-enriched pellets was ∼60% of the cortical pellets (Fig. 7*B*). These findings were consistent with previous work that indicated lack of correlation for overall tau concentration and seeding activity (29, 30).

**Figure 8:**
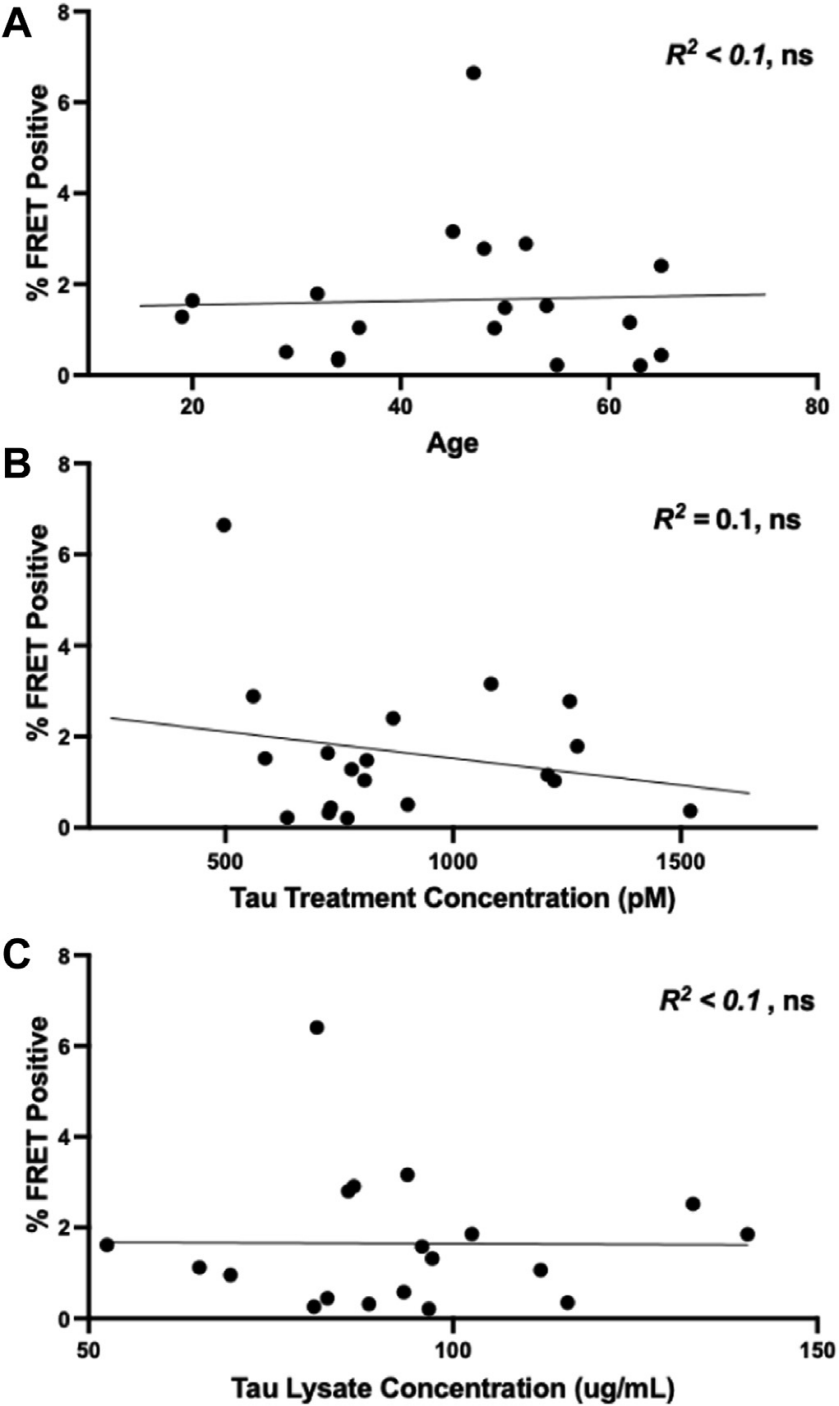
**Lack of correlation of tau seeding with age or tau concentration.***A*, initial tau concentration in total soluble protein fractions did not correlate with seeding in immunoprecipitation pellets. *B*, the final tau concentration from immunoprecipitation pellets did not correlate with seeding in immunoprecipitation pellets. *C*, age did not correlate with seeding in immunoprecipitation pellets. Data were analyzed using Pearson correlation, ns, not significant. FRET, fluorescence resonance energy transfer.

Age is the primary risk factor for the most common tauopathy, AD, and thus we tested for its correlation with control seeding activity. We observed no relationship between seeding activity and age (Fig. 8*A*). We further tested the correlation with seeding of tau levels in the pellets following IP (Fig. 8*B*) and total tau levels in human control total protein lysates (Fig. 8*C*) and also observed no correlation. We thus concluded that the seeding activity we observed in our cohort most likely did not represent an incipient age-dependent tauopathy, preferential tau pull-down, or increased tau levels.

### Seeds from controls *versus* AD exhibit distinct epitope accessibility

**Figure 9:**
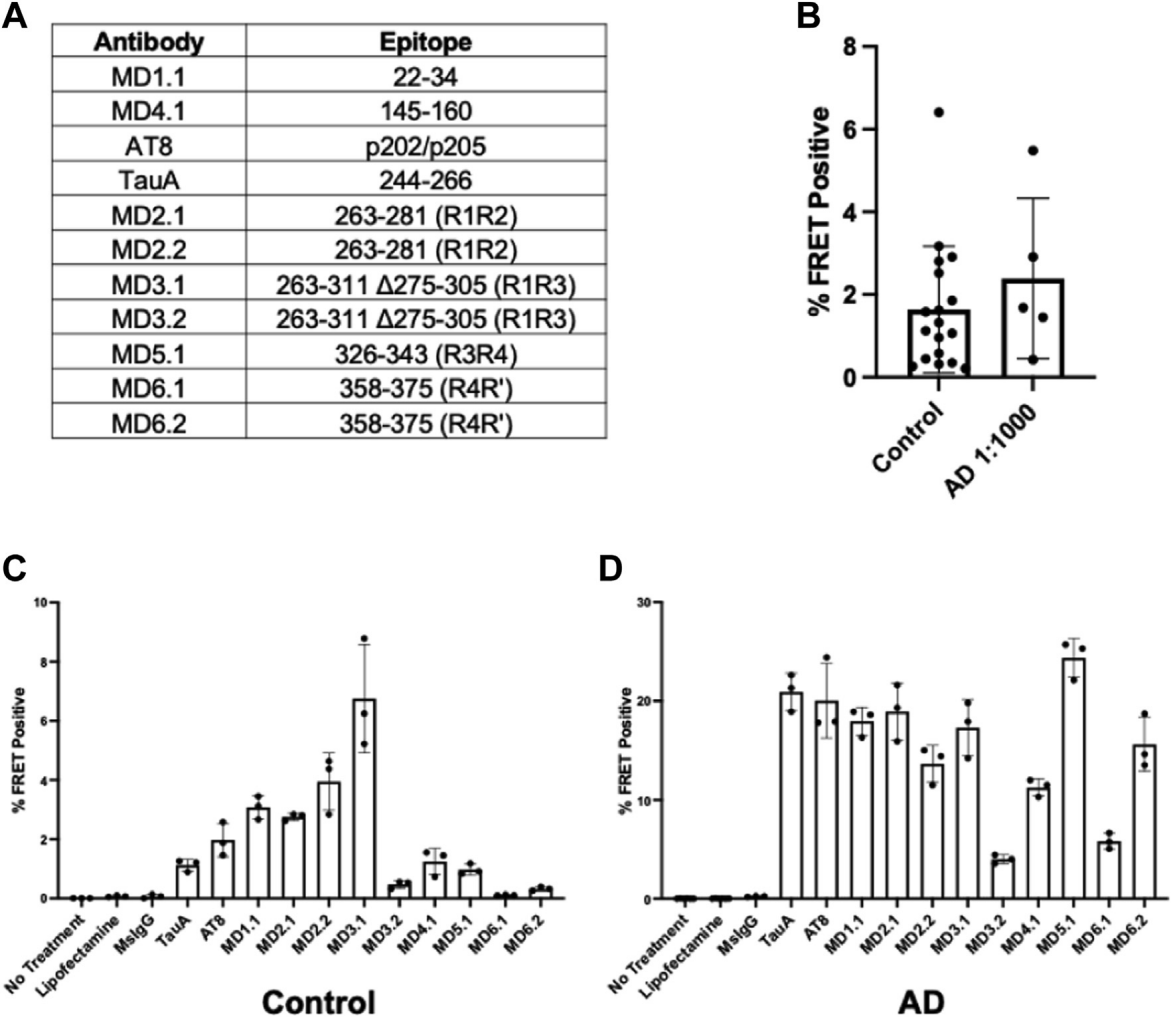
**Differential seed capture efficiency from control and Alzheimer’s disease (AD) brain.** A custom antibody panel reveals unique epitope exposure of tau seeds in control brain *versus* AD. *A*, epitopes of antibodies used. *B*, MD3.1 immunoprecipitation pellets from AD brain have roughly 1000× seeding activity *versus* MD3.1 pellets from control brain. MD3.1 pellets from AD were diluted 1000-fold prior to seeding on v2H biosensors while control pellets were used undiluted. No significant difference between undiluted control pellets and 1000-fold diluted AD pellets was found (*p* < 0.3692), Student’s *t* test, error bars = SD. (*C*) MD3.1 was most efficient at isolating tau seeds from control brain. *D*, multiple antibodies efficiently isolated seeds from AD brain. FRET, fluorescence resonance energy transfer.

Tau assemblies in different tauopathies exhibit conformational variation that can be resolved using cryo-EM. However, in the absence of significant amounts of insoluble tau it was impossible to determine the structure of the tau seeds we detected in control brains. Instead, we further tested the conformation of the seeds using a panel of antibodies raised against distinct epitopes across the tau protein (Fig. 9*A*). Based on dilution, MD3.1 immunoprecipitated from five distinct AD brains contained, on average, ∼1000x more seeding activity *versus* MD3.1 pellets from control brains (Fig. 9*B*). MD3.1 most efficiently immunoprecipitated tau seeds from control brain, while antibodies against R1 and more N-terminal residues more efficiently immunoprecipitated AD seeds *versus* antibodies directed against R3/R4 and more C-terminal residues (Fig. 9*C*). MD5.1, directed against residues of R3/R4, most efficiently precipitated AD seeds (Fig. 9*D*). Notably, compared with AD, MD6.2, directed against R4R′, inefficiently bound control seeds. Given the differential efficiencies in seed capture for the antibody panel, we concluded that control seeds likely represented a distinct strain or ensemble of strains *versus* AD.

## Discussion

In this study we have used a conformation-specific antibody and an ultrasensitive tau biosensor cell line to detect tau seeding in the parietal cortex in 19 control individuals with no known neurodegenerative or psychiatric diseases. Notably, we detected very low, or nonexistent, seeding within the cerebellums of these subjects and none in WT mice or knockin mice expressing the full human tau gene. The very low concentration of seeds precluded detailed structural analyses; however, an orthogonal seed amplification assay to detect tau seeds, RT-QuIC, confirmed the essential findings, albeit with lower sensitivity. We detected no AT8 immunoreactivity in control brain samples. Control brain seeding activity also did not correlate with tau level or subject age. Tau seeding was present in control brain at levels <1/1000 of AD brain and had different reactivity with a panel of anti-tau antibodies, suggesting that it was composed of tau conformers distinct from AD. These findings confirmed the existence of very low levels of tau seeds in healthy control human brain, in the absence of detectable tauopathy.

### Fidelity of tau seed detection

We have previously determined that P301S tau biosensors are specific for tau *versus* other common amyloid proteins such as a-synuclein and huntingtin (32). Likewise, RT-QuIC is highly specific for tau (34, 35, 36, 37). Interestingly, immunoenrichment with MD3.1 did not improve detection *via* RT-QuIC. This could be because contaminants in the brain homogenate have a greater effect on seeding from the biosensor assay or RT-QuIC detects a different seed population than MD 3.1. However, the high concordance between these two assays in positive samples and negative controls (mouse brain) indicated that, although overall seeding activity was low, it was highly likely to be specific and not due to “background” signal from independent seeding assays.

Importantly, the MD3.1 antibody used to isolate tau seeds did not induce a transition from inert to seed competence in recombinant tau monomers. We failed to detect strong tau seeding activity in biosensor cells in human cerebellum or hTau knockin mouse brain after tau immunopurification. If the experimental methods caused seed conversion, we would expect to find seeding activity in any immunopurified sample containing FL human tau. These results indicated that tau in healthy human brain adopts seed-competent conformations and this activity is specific to certain regions and cell types.

### Tau assemblies in normal brain function

The presence of tau seeds in healthy control brain might be expected if replication of unique assembly structures were linked to a normal function of tau. The precedent for functional prions exists as a mechanism of signaling in the immune system (21, 22) and at synapses (26). If amyloid formation were a critical aspect of tau’s normal biology, we would expect tau seeds to be present across all ages, and, indeed, we found clear, statistically significant seeding in 16/19 samples across a large age range. The absence of statistically significant seeding for three cases might relate to particular subregions of cortex that were sampled or postmortem interval. In contrast to individuals with tauopathy, age did not predict seeding activity. For example, the youngest individual with significant seeding was 19 years old, far too young to exhibit a sporadic tauopathy. Given the general paucity of healthy pediatric brain tissue we could not determine how early in life tau seeds appear. It remains to be determined what might be the functional role of tau seeds in healthy brain, although recent work from our laboratory suggests it could relate to RNA regulation (41). Indeed, multiple prior studies have indicated that RNA can induce tau seeding (42) and that RNA is associated with neurofibrillary tangles (43).

### Regional and species specificity of tau assembly formation

We observed a regional and species specificity for tau seeding. We detected seeding in the parietal cortex, minimal amounts in the cerebellum, and none in hTau knockin mouse cortex or cerebellum. This could reflect that tau forms no functional assemblies in these conditions or conversely that the strains formed were not efficiently precipitated by MD3.1 or amplified by RT-QuIC. This will require further study, and mechanisms controlling the formation and dissolution of tau seeds in the healthy brain will be critical to understand. If this process is dynamic, then it seems likely that human genetic factors, and even the state of brain function, might regulate seed abundance. Tau ligands or posttranslational modifications might also regulate seed levels. Pathogenic tau strains may thus represent perturbation of tau’s normal function, in which certain strains are inefficiently cleared or, combined with metabolic or genetic changes, lead to feed-forward loops of amplification.

### Control brain may harbor unique tau strains *versus* AD

One explanation for our findings is that control brain seeding here simply represents the earliest form of AD or some other tauopathy. We feel that undiagnosed tauopathy is highly unlikely in this case for several reasons. First, the cell-based biosensor assay we used is enormously sensitive to incipient tauopathy and indeed predicts pathology in humans or mice prior to the development of immunohistochemical evidence of disease using AT8 or similar antibodies (30, 32). Given that none of the samples studied showed any seeding in biosensors without immunoenrichment, we feel very confident that there was no undiagnosed tauopathy in the brain samples. Second, we observed no age dependence of control brain seeding, which would have been expected if it simply represented incipient sporadic tauopathy. Indeed, we observed relatively strong seeding in a 19-year-old subject, an age at which it would be essentially inconceivable to observe sporadic tauopathy. Third, we directly evaluated brain lysates using dot blot and immunohistochemistry for reactivity with AT8. We detected similar background AT8 signal in conditions where we observed seeding (parietal cortex) and where we did not (cerebellar cortex), in both cases at a level <1/10 to 1/100 of that detected in AD. Finally, using antibodies that target epitopes across tau, we observed different IP efficiencies for control *versus* AD seeds. It is possible that posttranslational modifications or associated cofactors led to differential IP, with the underlying seed conformation being unchanged. However, the simplest interpretation of our findings is that control seeds differ in epitope exposure because of their assembly structure, *i.e.*, strain identity.

In summary, we have detected tau seeds by IP in healthy brain in an age-independent, region-specific manner. The presence of seeds in healthy individuals suggests that tau forms self-replicating assemblies that may play a physiological role, rather than being purely pathological. Whether tau seeds have a functional role in healthy brain, and if critical cofactors or posttranslational modifications lead to dysregulated tau assembly formation, will require more investigation. This could be especially important in light of therapies to clear tau assemblies, or inhibit their formation, which might have unintended consequences for normal brain function.

## Experimental procedures

### Biosensor cell line v2H

Highly sensitive second-generation tau biosensor cells termed v2H (28) were used for seeding assays. These cells are based on expression of tau repeat domain fragment (246–378) containing the disease-associated P301S mutation (tau-RD) fused to mCerulean3 or mClover3. The v2H line was selected for high expression with low background signal and high sensitivity. Seeding experiments used established protocols (32).

### Cell culture

v2H biosensors were grown in Dulbecco’s modified Eagle’s medium (Gibco) supplemented with 10% fetal bovine serum (HyClone) and 1% glutamax (Gibco). For terminal experiments, 1% penicillin/streptomycin (Gibco) was included. Cells were tested free of mycoplasma (VenorGem, Sigma) and cultured at 37 °C with 5% CO_2_ in a humidified incubator. To avoid false-positive signal from v2H biosensors, cells were passaged prior to ∼80% confluency.

### Human brain samples

Human brain tissue was obtained from 19 control subjects (6 females, 13 males, age range 19–65 years, Table 1) without any known tauopathy or psychiatric diagnoses, with Institutional Review Board approval at University of Texas Southwestern Medical Center. The AD case used for immunohistochemistry and biochemistry derived from a female, age 84 years, Braak stage VI; the other cases used for biochemistry were from the following patients, all Braak stage VI: male, age 73 years; female, age 74 years; male, age 76 years; male, age 82 years; female, age 83 years. Informed written consent for donation of tissue was obtained from next of kin prior to collection. Brains were sectioned and flash frozen in liquid nitrogen for long-term storage at −80 °C. Pulverized frozen tissue from the cortex of the parietal lobe and cerebellum was used to prepare total soluble protein lysates for further experiments. Brain tissue from a deceased, de-identified case of sporadic Alzheimer’s disease (36) was kindly provided by Prof Bernardino Ghetti of the Dementia Laboratory at Indiana University School of Medicine for use in the RT-QuIC assay.

### Human sample preparation

Fresh-frozen pulverized tissue was suspended in Tris-buffered saline (TBS) containing cOmplete mini protease inhibitor tablet (Roche) at a concentration of 10% w/vol. Samples were then Dounce homogenized, followed by pulsing probe sonication at 75 W for 10 min (Q700, QSonica) on ice in a ventilated hood. The sonication probe was washed with a sequence of ethanol, bleach, and distilled water to prevent cross-contamination. Lysates were then centrifuged at 23,000*g* for 30 min, and the supernatant was retained as the total soluble protein lysate. Protein concentration was measured with the bicinchoninic acid assay (Pierce). Fractions were aliquoted and stored at −80 °C prior to IP and seeding experiments.

### Mouse lines

Tau KO mice (n = 10, 5 females, 5 males, average age 12.3 months) containing a GFP-encoding cDNA integrated into exon 1 of the MAPT gene were negative controls (44). Tau KO mice were obtained from Jackson Laboratory and maintained on a C57BL/6J background. Wildtype mice (n = 9, 5 females, 4 males, average age 12.9 months) of BL6/C3H background were used as a source of murine tau. We used mice on BL6 background with the human MAPT gene knocked in at the mouse tau locus that express all six isoforms of human tau (n = 10, 5 females, 5 males, average age 16.2 months) as a source of murine-expressed human tau (gift from Pfizer), and verified by genomic sequencing in the Diamond lab. All mice involved in this study were housed under a 12-h light/dark cycle and were provided food and water *ad libitum*. All experiments involving animals were approved by the University of Texas Southwestern Medical Center Institutional Animal Care and Use Committee (IACUC).

### Mouse sample collection and preparation

Mice were anesthetized with isoflurane and perfused with chilled phosphate-buffered saline (PBS) + 0.03% heparin. The forebrain and cerebellum were separated and weighed prior to flash freezing in liquid nitrogen and storage at −80 °C. As described previously for human tissues, fresh-frozen forebrain was suspended in TBS containing cOmplete mini protease inhibitor tablet (Roche) at a concentration of 10% w/vol. Samples were then Dounce homogenized, followed by pulsing probe sonication at 75 W for 10 min on ice in a hood (Q700, QSonica). The sonication probe was washed with a sequence of ethanol, bleach, and distilled water to prevent cross-contamination of seeding activity. Lysates were then centrifuged at 23,000*g* for 30 min, and the supernatant was retained as the total soluble protein lysate. Protein concentration was measured with the bicinchoninic acid assay (Pierce). Fractions were aliquoted and stored at −80 °C prior to IP and seeding experiments.

### Immunoprecipitation from protein lysate or tau monomer

IPs were performed using 50 μl of magnetic Protein A Dynabead slurry (ThermoFisher), washed twice with IP wash buffer (0.05% Triton-X100 in PBS), followed by a 1-h room temperature incubation with 20 μg of anti-tau antibody. Beads were washed 3 times in IP wash buffer; 1000 μg of total protein lysate or 500 ng of recombinant tau monomer was added to the Protein A/anti-tau antibody complexes on the beads and rotated overnight at 4 °C. After overnight incubation, the supernatant was removed as the tau-depleted fraction and the beads were washed 3 times in IP wash buffer and then moved to clean tubes for elution. The IP wash buffer was removed, and beads were then incubated in 65 μl of IgG Elution Buffer (Pierce) for 7 min to elute tau. The elution buffer was collected in a separate microcentrifuge tube, and a second elution step in 35 μl of IP elution buffer was performed for 5 min and pooled with the initial elution. The elution was then neutralized with 10 μl of Tris-HCl pH 8.4 to finalize the tau-enriched IP pellet.

### Enzyme-linked immunosorbent assay for total tau

A total tau “sandwich” ELISA was performed as described (40). Anti-tau antibody reagents were kindly provided by the late Dr Peter Davies (Albert Einstein College of Medicine). Ninety-six-well round-bottom plates (Corning) were coated for 48 h at 4 °C with DA-31 (aa 150-190) diluted in sodium bicarbonate buffer (6 μg/ml). Plates were rinsed with PBS 3 times, blocked for 2 h at room temperature with Starting Block (Pierce), and rinsed with PBS five additional times. Total protein was diluted 1:1000 in SuperBlock solution (Pierce; 20% SuperBlock in TBS), and 50 μl sample was added per well. Tau-enriched IP pellets were diluted 1:100 in SuperBlock solution, and 50 μl sample was added per well. DA-9 (raised against aa 102-150) was conjugated to HRP using the Lightning-Link HRP Conjugation Kit (Innova Biosciences), diluted 1:50 in superblock solution, and 50 μl was added per well (15 μg/ml). Sample and detection antibody complexes were incubated overnight at 4 °C. Plates were then washed with PBS 9 times with a 15 s incubation between each wash, and 75 μl 1-Step Ultra TMB Substrate Solution (Pierce) was added. Plates were developed for 30 min, and the reaction was quenched with 2 M sulfuric acid. Absorbance was measured at 450 nm using an Epoch plate reader (BioTek). Each plate contained a standard curve, and all samples were run in duplicate. Tau concentration was calculated using MyAssays four parameter logistic curve fitting tool.

### Dot blot assays

Soluble protein lysates were prepared as described previously. Lysates were diluted to a final total soluble protein concentration of 1 mg/ml in TBS containing cOmplete mini protease inhibitor tablet (Roche). For AD, serial dilutions were subsequently prepared. Two microliters per sample was spotted onto nitrocellulose membrane (ThermoFisher Scientific) and then blocked in StartingBlock (TBS) blocking buffer (ThermoFisher Scientific) for 30 min at room temperature. After blocking, the blot was incubated overnight at 4 °C with AT8 primary antibody (Invitrogen) diluted 1:1000 in blocking buffer. The membrane was washed in TBS-T 5 times for 5 min followed by 1 h room temperature incubation with peroxidase-conjugated anti-mouse secondary antibody (Jackson ImmunoResearch) diluted 1:4000 in blocking buffer. The membrane was then washed an additional 5 times in TBS-T. The membrane was developed with ECL Prime Western blot detection kit (Cytiva) and imaged with a Syngene digital imager.

### Transduction of biosensor cell lines, flow cytometry, and seeding analyses

The seeding assay was conducted as described (32) with the following changes: v2H cells were plated 20 h before seed transduction at a density of 16,000 cells/well in a 96-well plate in a medium volume of 180 μl per well. Mouse and human total protein lysates were thawed on ice, while tau-depleted IP supernatants and tau-enriched IP pellets were isolated just before seeding. For total protein lysates and tau-depleted supernatants 10 μg of protein was used per well. For tau-enriched pellets, 10 μl of elution was used per well. Samples were incubated for 30 min with 0.5 μl Lipofectamine 2000 (Invitrogen) and OptiMEM such that the total treatment volume was 20 μl. For each experiment, cells treated with OptiMEM alone and Lipofectamine 2000 in OptiMEM were used as negative controls. The v2H line, which expresses high levels of tau-RD, can show false-positive fluorescence resonance energy transfer (FRET) signal when treated with Lipofectamine 2000, which is mitigated by passaging prior to ∼80% confluency. Recombinant tau fibrils at 1 pM and 100 fM (monomer equivalent) were used for positive controls. Cells were incubated for an additional 48 h after treatment prior to harvesting. Cells were harvested with 0.25% trypsin and fixed in 4% paraformaldehyde for 10 min, then resuspended in flow cytometry buffer (Hanks' balanced salt solution plus 1% fetal bovine serum and 1 mM EDTA). The LSRFortessa SORB (BD Biosciences) was used to perform FRET flow cytometry. Single cells double-positive for mCerulean and mClover were identified and the % FRET-positive cells within this population was quantified following a gating strategy described (32). For each experiment 10,000 cells were analyzed in triplicate. Flow data analysis was performed using FlowJo v10 software (Treestar).

### RT-QuIC assay

The K12CFh substrate was purified, and the K12 RT-QuIC assay was performed as detailed in Metrick *et al.* (36). Coded aliquots of total homogenates, supernatants, and immunoprecipitated pellets were thawed on ice and serially diluted in a buffer containing 1× N-2 Supplement (Gibco) in 10 mM Hepes and 0.53% tau knockout mouse brain homogenate (KO; B6.129S4(Cg)-Mapttm1(EGFP)Klt/J from Jackson Laboratories). Sporadic AD frontal cortex brain homogenate was prepared as described in Metrick *et al*. (36). The tissue was homogenized using a Beadbeater (Biospec) with 1-mm silica beads (BioSpec, 11079110z) in ice-cold 1 × PBS, pH 7.4. The K12CFh substrate was thawed in room temperature and filtered using a 100 kDa spin filter at 6000 rpm for 5 min. The reaction mix was prepared containing 40 mM Hepes, 400 mM NaF, 40 μM heparin, and 10 μM thioflavin T (ThT) and the K12CFh substrate was added at a final concentration of 6.5 μM (∼0.1 mg/ml). All reactions were performed in a 384-well optical BTM Polybase Black plate (NUNC) and contained 48 μl of reaction mix, seeded with 2 μl of the diluted sample (*i.e.*, total, sup, pellet) for a total volume of 50 μl per well. All samples were assayed with two different K12CFh substrate batches (except for one sample Cortex Sup S), using quadruplicate technical replicate reactions per substrate batch. The plates were sealed with sealing tape and placed in an Omega FLUOStar plate reader. The RT-QuIC assay was performed at 42 °C with intervals of 1 min shaking at 500 rpm and 1 min rest. ThT fluorescence signals were collected in either 15- or 45-min intervals, at an excitation wavelength of 450 nm and an emission wavelength of 480 nm. Reactions were considered positive if the ThT fluorescence signal exceeded the threshold, calculated as the average of baseline readings plus 100 times the standard deviation of those readings. Data analysis was performed with BMG Labtech Mars Omega (v3.32), GraphPad Prism (v9.3.1), and Microsoft Excel.

### Isolation of mouse brain

Animals were anesthetized with isoflurane and perfused with cold PBS. Brains were hemi-dissected. The right hemispheres were frozen in liquid nitrogen and stored at −80 °C for subsequent biochemical assays while the left hemispheres were drop-fixed in Phosphate-Buffered 4% Paraformaldehyde (FD NeuroTechnologies) overnight at 4 °C. Left hemispheres were then placed in 10% sucrose in PBS for 24 h at 4 °C, followed by 24 h in 20% sucrose in PBS at 4 °C, and finally stored in 30% sucrose in PBS at 4 °C until sectioning.

### Immunohistochemistry

A sliding-base freezing microtome (ThermoFisher Scientific) was used to collect 40 μm free-floating sagittal sections from the fixed mouse and human brains. Sections were stored in cryoprotectant at 4 °C until immunohistochemisty was performed. Slices were first blocked for 1 hour with 5% bovine serum albumin in TBS with 0.25% Triton X-100 (blocking buffer). Brain slices were incubated with biotinylated AT8 antibody (1:500, Thermo Scientific) overnight in blocking buffer at 4 °C. Slices were subsequently incubated with the VECTASTAIN Elite ABC Kit (Vector Labs) in TBS prepared according to the manufacturer’s protocol for 30 min, followed by DAB development using the DAB Peroxidase Substrate Kit (Vector Labs). Slices were imaged using the Olympus Nanozoomer 2.0-HT (Hamamatsu) in the University of Texas Southwestern Medical Center Whole Brain Microscopy Core Facility (RRID: SCR_017949).

### Statistical analyses

Coded samples were obtained by M. S. L. from the lab of C. A. T. M. S. L. remained blinded prior to all seeding analyses. Flow cytometry gating and analysis of seeding activity was completed prior to decoding and interpreting the results. All statistical analysis was performed using GraphPad Prism v9.2.0 for Mac OS and Excel v16.52 (Microsoft).

## Data availability

Data described in this article is contained within. All raw data flow cytometry data can and will be provided by M. S. L. (Michael.lacroix@utsouthwestern.edu) upon request.

## Supporting information

This article contains supporting information.

## Conflict of interest

The authors declare that they have no conflicts of interest with the contents of this article.

## Supporting information

Supporting Information Figure 1 **MD3.1 antibody does not induce seed conversion of recombinant tau monomer under experimental immunoprecipitation conditions.** No seeding activity was detected in the pellet after immunoprecipitation of 500 ng recombinant 2N4R tau with MD3.1. Statistical significance was determined by performing one-way ANOVA followed by Tukey’s multiple comparisons test, ∗*p* < 0.05, ∗∗*p* < 0.01, ∗∗∗*p* < 0.001, ∗∗∗∗*p* < 0.0001. Errors bars = S.D.

Supporting Information Figure 2 **Western blots for human tau expression in hTau mice.***A*, HJ8.5, a human tau specific antibody recognizing n-terminal residues 25–30, shows human tau expression in Pfizer human tau mice. No human tau is detected in WT mice, or tau knockout mice used as a negative control. *B*, low exposure of Dako polyclonal tau antibody reveals tau expression in hTau and WT mice, human and mouse, respectively. High exposure reveals non-specific bands in knock-out mice.

Supporting Information Figure 3 **ThT fluorescence maxima for representative control cortex samples.** Mean ThT fluorescence maxima obtained for 10^−3^ dilutions of representative cortex samples relative to those of negative [unseeded & tau-free (KO) mouse] and positive (sporadic Alzheimer’s disease) controls illustrating the distinction between non-tauopathy cortex specimens and tau-free controls. As expected, these means are lower than those obtained for the sAD positive control brain.
