# Supplementary figures and images for: Tau seeding without tauopathy
Source: J Biol Chem. 2023 Dec 9;300(1):105545. doi: 10.1016/j.jbc.2023.105545 (PMC10797195; doi:10.1016/j.jbc.2023.105545)

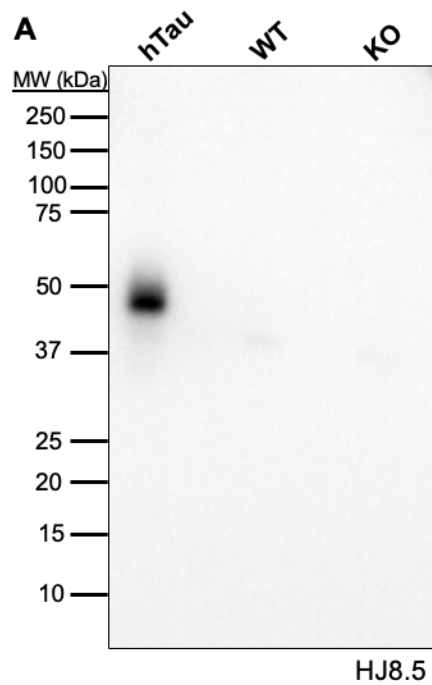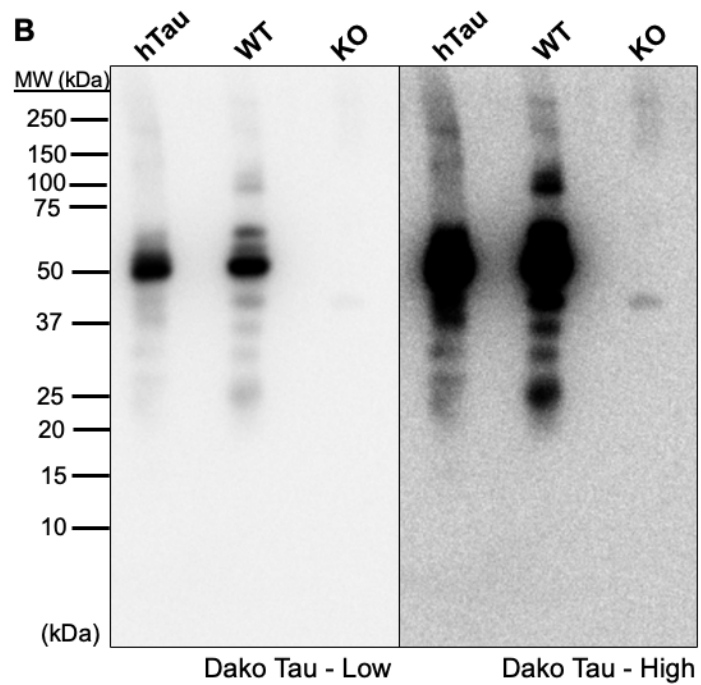

Supplement: Supporting Information Figure 1 — MD3.1 antibody does not induce seed conversion of recombinant tau monomer under experimental immunoprecipitation conditions. No seeding activity was detected in the pellet after immunoprecipitation of 500 ng recombinant 2N4R tau with MD3.1. Statistical significance was determined by performing one-way ANOVA followed by Tukey’s multiple comparisons test, ∗p < 0.05, ∗∗p < 0.01, ∗∗∗p < 0.001, ∗∗∗∗p < 0.0001. Errors bars = S.D. [file mmc1.pdf]

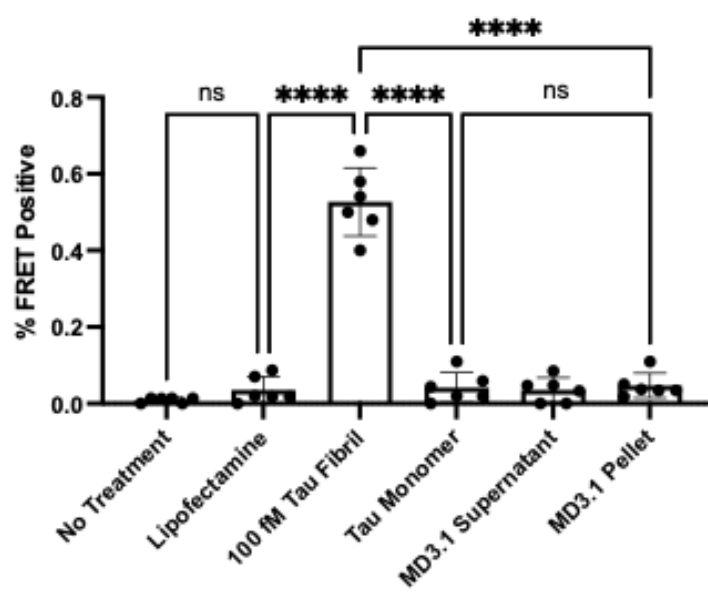

Supplement: Supporting Information Figure 2 — Western blots for human tau expression in hTau mice.A, HJ8.5, a human tau specific antibody recognizing n-terminal residues 25–30, shows human tau expression in Pfizer human tau mice. No human tau is detected in WT mice, or tau knockout mice used as a negative control. B, low exposure of Dako polyclonal tau antibody reveals tau expression in hTau and WT mice, human and mouse, respectively. High exposure reveals non-specific bands in knock-out mice. [file mmc2.pdf]

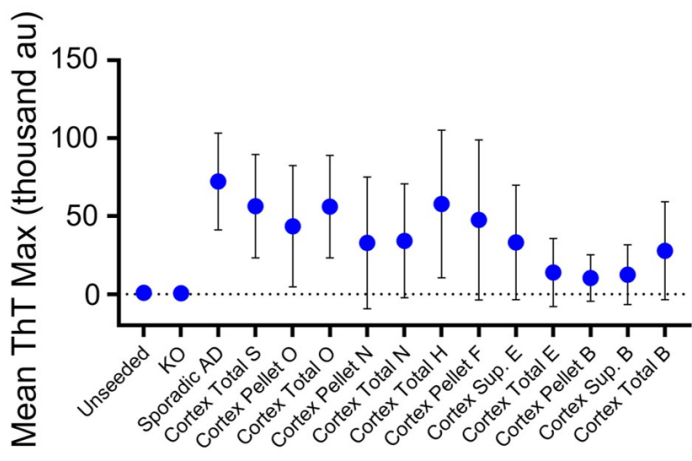

Supplement: Supporting Information Figure 3 — ThT fluorescence maxima for representative control cortex samples. Mean ThT fluorescence maxima obtained for 10−3 dilutions of representative cortex samples relative to those of negative [unseeded & tau-free (KO) mouse] and positive (sporadic Alzheimer’s disease) controls illustrating the distinction between non-tauopathy cortex specimens and tau-free controls. As expected, these means are lower than those obtained for the sAD positive control brain. [file mmc3.pdf]
